# The Impact of a Multidisciplinary Residential Program (MRP) on Body Composition, Psychological Well-Being, and Hematochemical Parameters in Hospitalized Obese Patients

**DOI:** 10.3390/nu17132108

**Published:** 2025-06-25

**Authors:** Simone Perna, Giuseppe Mazzola, Michela Seniga, Gaetan Claude Barrile, Ilaria Torello, Alessia Moroni, Francesca Mansueto, Alessandro Lazzarotti, Vai Veronica, Clara Gasparri, Mariangela Rondanelli

**Affiliations:** 1 Department of Food, Environmental and Nutritional Sciences, Division of Human Nutrition, University of Milan, 20133 Milan, Italy; simone.perna@unimi.it; 2 Endocrinology and Nutrition Unit, Azienda di Servizi alla Persona “Istituto Santa Margherita”, University of Pavia, 27100 Pavia, Italy; michela.seniga01@universitadipavia.it (M.S.); gaetanclaude.barrile01@universitadipavia.it (G.C.B.); ilaria.torello@gmail.com (I.T.); alessia.moroni02@universitadipavia.it (A.M.); francesca.mansueto01@universitadipavia.it (F.M.); alessandro.lazzarotti01@universitadipavia.it (A.L.); veronica.vai.2001@gmail.com (V.V.); clara.gasparri01@universitadipavia.it (C.G.); 3 Department of Public Health, Experimental and Forensic Medicine, University of Pavia, 27100 Pavia, Italy; mariangela.rondanelli@unipv.it

**Keywords:** obesity, multidisciplinary intervention, body composition, psychological well-being, biochemical parameters, hospitalized patients, nutritional therapy, physical activity, mental health, obesity treatment strategies

## Abstract

**Introduction**: Obesity is a multifactorial condition strongly associated with physical and psychological comorbidities. This study aimed to evaluate changes in psychological symptoms and their correlation with anthropometric and body composition improvements in hospitalized obese patients undergoing a multidisciplinary rehabilitation program (MRP). **Methods**: A total of 178 obese patients (61 males and 117 females; mean age 58.5 ± 14.0 years; mean BMI 41.3 ± 6.1 kg/m^2^) completed a two-month structured intervention combining a low-energy Mediterranean-style diet, individualized physical activity, and psychological support. Body composition by DXA, biochemical markers, and psychological outcomes (Beck Depression Inventory [BDI] and Binge Eating Scale [BES]) were assessed at baseline and discharge. **Results**: At baseline, 72.3% of participants showed depressive symptoms (BDI > 10), and 42.7% exhibited binge eating behaviors (BES ≥ 17). The intervention led to significant reductions in weight (−7.08 kg), BMI (−2.68 kg/m^2^), fat mass (−4.43 kg), and visceral adipose tissue (−329 g) (all *p* < 0.001). Mean BDI and BES scores decreased by 5.9 and 6.4 points, respectively (both *p* < 0.001). Moderate correlations were observed between reductions in adiposity and improvements in psychological symptoms, such as r = −0.45 for depressive symptoms (BDI) and r = −0.39 for binge eating behaviors (BES) (*p*-values < 0.001). **Conclusions**: A structured multidisciplinary intervention significantly improved body composition and psychological well-being in hospitalized obese patients. The moderate association between reduced adiposity and alleviation of depressive symptoms and binge eating behaviors underlines the value of integrated physical, nutritional, and psychological care. **Level of Evidence**: Level 3, according to the Oxford Centre for Evidence-Based Medicine.

## 1. Introduction

Obesity is a complex condition resulting from the interplay of genetic, environmental, behavioral, and psychosocial factors, and it is associated with a wide range of cardiovascular, respiratory, musculoskeletal, metabolic, and psychosocial complications, necessitating a multidisciplinary approach for effective management [1]. Globally, obesity represents a major public health challenge, with recent estimates indicating that over 650 million adults and 340 million children and adolescents are affected [2].

Beyond its somatic consequences, obesity is strongly linked to mental health disorders, including depression, anxiety, and binge eating disorder, forming a bidirectional relationship where obesity exacerbates psychiatric conditions and vice versa [3,4,5]. Stigmatization and impaired body image perception further compound the psychological burden in individuals with obesity [6,7]. Importantly, the prevalence of depressive symptoms among individuals with obesity remains high even after adjusting for demographic and health factors. Although the hypothesis that obesity directly leads to psychiatric, psychological, or neurological conditions remains debated, it is widely accepted that obesity is associated with a range of biomolecular changes. Shared biological pathways—including chronic inflammation, oxidative stress, and hypothalamic–pituitary–adrenal axis dysregulation—partially explain the overlap between obesity and psychiatric conditions [7,8,9]. Psychological stressors such as body dissatisfaction and social isolation further fuel maladaptive behaviors, including emotional eating and physical inactivity, reinforcing the obesity cycle [6,7].

Hospital-based multidisciplinary rehabilitation programs offer structured environments conducive to achieving both anthropometric improvements and psychological benefits. These settings allow for close clinical monitoring, professional supervision, and higher adherence rates compared to outpatient interventions. Several studies suggest that weight loss interventions significantly reduce depressive and anxiety symptoms [6,7]. However, most existing research has assessed these domains separately, with limited integration of body composition metrics, biochemical parameters, and psychological outcomes. Despite growing interest in the multidisciplinary treatment of obesity, few studies have evaluated psychological, body composition, and biochemical outcomes in an integrated manner. Most of the existing literature tends to address these domains separately, limiting the understanding of their interrelations within clinical practice.

The primary aim of this study was to evaluate changes in psychological symptoms and their association with anthropometric, body composition, and biochemical improvements in a large cohort of hospitalized obese patients undergoing a multidisciplinary residential program (MRP). The MRP applied in this study corresponds to the institutional multidisciplinary protocol adopted at the “Santa Margherita” Institute in Pavia (Italy), which has already been examined in its most updated versions in previous publications [10,11]. This research provides a comprehensive real-world evaluation by integrating DXA-measured body composition, biochemical markers, and validated psychological assessments within a single inpatient cohort, addressing an important gap in the current literature.

## 2. Materials and Methods

### 2.1. Study Design and Setting

This prospective, open-label observational cohort study was conducted at the Metabolic Rehabilitation Unit of the Azienda di Servizi alla Persona, Istituto Santa Margherita, University of Pavia (Pavia, Italy). Both participants and investigators were aware of the intervention protocol. The study was approved by the Ethics Committee of the University of Pavia, and all participants provided written informed consent prior to enrollment.

Data were collected between 1 January 2016 and 1 March 2021, in accordance with the CONSORT guidelines for observational studies [12]. The multidisciplinary residential program (MRP) was delivered in a fully supervised inpatient setting and lasted up to a maximum of three months, with individual durations ranging from two to approximately three months depending on clinical needs and recommendations from the multidisciplinary care team. Patients who discontinued the program early or stayed for a period considered insufficient to complete the planned intervention were excluded from the final analysis. Outcomes were assessed at baseline (T0) and at the end of the recovery period (duration of hospitalization) (T1).

### 2.2. Multidimensional Residential Program (MRP)

#### 2.2.1. Nutritional Intervention

The nutritional intervention consisted of a low-energy mixed diet providing approximately 50–55% of total energy from carbohydrates, 25–30% from lipids, and 15–20% from proteins. The diet was individually prescribed to achieve an energy deficit of approximately 500–600 kcal/day, based on the patient’s total energy expenditure (TEE), which was calculated using measured resting energy expenditure (REE) and estimated physical activity level (PAL), in line with clinical recommendations [9] The total energy expenditure was calculated by multiplying the resting energy expenditure (REE), measured by indirect calorimetry (Quark RMR, Cosmed, Rome, Italy) for 30 min in the morning with patients in a fasting state (≥12 h) under controlled ambient conditions (temperature 22–24 °C, dim light, and quiet environment), by a physical activity level (PAL) estimated according to standardized procedures for energy requirement assessment, as recommended by international guidelines [13] and recent reviews on energy balance [14]. PAL was assigned based on the type, frequency, and duration of supervised exercise sessions performed during hospitalization, in agreement with the physiotherapy team. The dietary protocol adhered to the American Diabetes Association guidelines to ensure a safe weight loss of 0.5–1 kg per week and adequate protein intake for individuals with obesity [9].

REE was assessed via indirect calorimetry using a Deltatrac Monitor II MBM-200 (Datex Engstrom Division, Instruments Corp., Helsinki, Finland), following standardized measurement protocols. REE was derived from oxygen consumption and carbon dioxide production, as well as urinary nitrogen excretion, applying the Weir equation and expressed as kcal/day. In addition, the postprandial respiratory quotient (RQ) and substrate oxidation were evaluated through continuous gas exchange monitoring [15].

Individualized meal plans were designed by registered dietitians to match each participant’s metabolic requirements and food preferences. To maximize adherence, weekly one-on-one dietary counseling sessions were conducted, including weight monitoring, nutritional assessment, portion size guidance, and behavioral strategies aimed at promoting long-term compliance with the dietary regimen.

#### 2.2.2. Physical Activity

The physical activity intervention followed the World Health Organization’s recommendations for adults [16] and consisted of a concurrent training protocol combining aerobic and resistance exercises [17].

Due to the lack of definitive consensus on optimal exercise protocols in adults with severe obesity (body mass index [BMI] ≥ 40 kg/m^2^), this approach was selected based on evidence supporting its efficacy in improving metabolic and functional outcomes.

Each participant engaged in a 60 min supervised exercise session five days per week, including approximately 20–25 min of aerobic activities (such as treadmill walking or cycling) performed at moderate intensity (55–70% of heart rate reserve or RPE 12–14) and 20–25 min of resistance exercises (including bodyweight and elastic band exercises) targeting both upper- and lower-body muscle groups in 2–3 sets of 10–15 repetitions. All activities were tailored to the individual’s fitness level and progressively adapted over the course of the intervention.

In addition, participants were encouraged to reach a daily target of ≥10,000 steps as measured by a pedometer.

All exercise sessions were conducted under the supervision of licensed physiotherapists with specialized training in obesity rehabilitation.

#### 2.2.3. Behavioral and Psychodynamic Treatment

Psychological support was delivered using a multidimensional and standardized protocol designed for patients with obesity. At both baseline and discharge, participants completed the Beck Depression Inventory (BDI) [18,19], the State–Trait Anxiety Inventory (STAI) [20,21], the Penn State Worry Questionnaire (PSWQ) [22,23], the Binge Eating Scale (BES) [24,25], and the Body Uneasiness Test (BUT) [26,27]. These questionnaires were completed by patients during dedicated one-on-one sessions with licensed clinical psychologists, ensuring standardized administration and assisting comprehension of the questionnaire items.

All psychological interventions were delivered by licensed clinical psychologists and included weekly individual and group psychodynamic counseling sessions, based on validated therapeutic frameworks. The sessions aimed to increase emotional awareness, reduce stress, address maladaptive eating behaviors, and improve body image perception. Group therapy fostered peer support, emotional regulation, and the development of coping strategies to promote long-term adherence. Psychotherapy protocols were adapted to patient needs but followed standardized core principles to ensure consistency across the cohort.

### 2.3. Biochemical Analysis

Blood samples were collected at baseline and at the end of the intervention. The following biochemical parameters were assessed: serum iron, uric acid, creatinine, and total calcium, measured by enzymatic–colorimetric assay (Abbott Laboratories, Abbott Park, IL, USA); erythrocyte sedimentation rate (ESR), measured by the Westergren method using a Diesse Analyzer; blood electrolytes, measured by indirect ion-selective electrode (ISE) potentiometry (Abbott Laboratories); ionized calcium, measured by selective electrode potentiometry; insulin, measured by electrochemiluminescence immunoassay (ECLIA; Roche Diagnostics); and complete blood count (CBC), determined by differential blood cell counting. Insulin resistance was calculated using the homeostasis model assessment (HOMA) method [28].

### 2.4. Anthropometric Measurements

Anthropometric parameters, such as body weight, waist circumference, and hip circumference were measured weekly during the recovery period. Body weight was measured to the nearest 0.1 kg, using a precision scale; participants wore light clothing, no shoes, and a standardized method was used. The waist was measured at the midpoint between the top of the hip bone (iliac crest) and lowest rib, using a standardized method. Hip circumference was measured at the level of the widest portion of the buttocks, using a non-stretchable tape with the patient standing erect and feet together.

### 2.5. Body Composition Assessment by Dual-Energy X-Ray Absorptiometry (DXA)

Body composition (fat free mass, fat mass, and visceral fat mass) was determined by dual-energy X-ray absorptiometry (DXA), using a Lunar Prodigy DXA (GE Medical Systems, Chicago, IL, USA). In vivo CVs were 0.89% for whole body fat (fat mass) and 0.48% for fat-free mass (FFM). Whole body and fat free mass (FFM) were divided by height squared to obtain the FFM index (FFMI). FFM depletion was defined as having a whole-body FFMI below the 5th centile for age- and gender-matched healthy subjects [29]. Visceral adipose tissue (VAT) volume was estimated using a constant correction factor (0.94 g/cm^3^). The software automatically places a quadrilateral box, which represents the android region, outlined by the iliac crest and with a superior height equivalent to 20% of the distance from the top of the iliac crest to the base of the skull [30]. Subcutaneous abdominal fat was defined as the difference between android fat and visceral fat. The in vivo CVs were 0.89% and 0.48% for FM and FFM, respectively [31].

### 2.6. Statistical Analysis

Baseline and post-intervention data were analyzed to assess changes in key demographic, clinical, anthropometric, biochemical, and psychological variables. Paired *t*-tests were used to evaluate pre–post differences in continuous variables within the overall sample. Comparisons between groups, such as male vs. female participants, were conducted using Welch’s *t*-test to account for unequal group sizes and heterogeneity of variances. One-way ANOVA was employed for subgroup analyses involving multiple factors, including age categories and recovery period duration.

Normality assumptions were verified using the Shapiro–Wilk test. In the presence of non-normal distributions, appropriate data transformations or non-parametric alternatives were considered, although the primary analyses remained based on parametric tests due to sample size robustness. Effect sizes (Cohen’s d for *t*-tests and eta squared for ANOVA) were also calculated to estimate the magnitude of differences.

Correlation analyses were performed using Pearson’s coefficient to explore associations between changes in body composition, psychological outcomes, and biochemical markers. Only statistically significant correlations (*p* < 0.05) were reported in the graphical output. All statistical analyses were carried out using IBM SPSS Statistics for Windows, Version 28.0 (IBM Corp., Armonk, NY, USA), with a two-sided significance threshold of *p* < 0.05.

### 2.7. Ethical Considerations

The study protocol was conducted in accordance with the Declaration of Helsinki and approved by the Ethics Committee of the University of Pavia, Italy (approval number 6723/22052019). All participants provided written informed consent before enrollment. Patient confidentiality was ensured through anonymization of data, secure electronic storage, and restricted access to study personnel.

## 3. Results

Table 1 shows descriptive statistics of the sample at baseline, which refer to patients hospitalized from 1 January 2016 to 1 March 2021. The anthropometric characteristics of the patients at baseline are shown in Table 1. The sample included 42 males and 136 females, with an average age of 58.7 ± 12.7 years. The study included 178 adult patients (61 males and 117 females) with severe obesity and obesity-related comorbidities, admitted to the Metabolic Rehabilitation Program (MRP). The average age of the participants was 58.48 ± 13.97 years, with significant differences between males (54.49 ± 15.17) and females (61.69 ± 12.51, *p* ≤ 0.001). Anthropometric measures at baseline revealed that BMI was comparable across genders (41.34 ± 5.43 kg/m^2^ in males vs. 41.24 ± 6.52 kg/m^2^ in females, *p* = 0.904).

Body composition analysis showed males had higher fat-free mass (63.70 ± 7.65 kg vs. 46.04 ± 6.18 kg, *p* ≤ 0.001) and a lower fat mass percentage (43.59 ± 4.83% vs. 51.46 ± 4.76%, *p* ≤ 0.001) compared to females. Waist circumference, commonly used as a surrogate marker of abdominal fat distribution, was also significantly higher in males (132.64 ± 11.31 cm vs. 119.53 ± 12.54 cm, *p* < 0.001). These differences highlight the diverse physiological profiles within the cohort. The tables provide a summary of the study’s findings at baseline and after the multidisciplinary intervention. Table 1 and Table 2 describe the baseline and pre–post-intervention anthropometric, psychological, and biochemical characteristics of the sample, highlighting differences between males and females in body composition, psychological scores, and biochemical values. (Table 2 report values adjusted for age, recovery period, and gender).

**Figure 1:**
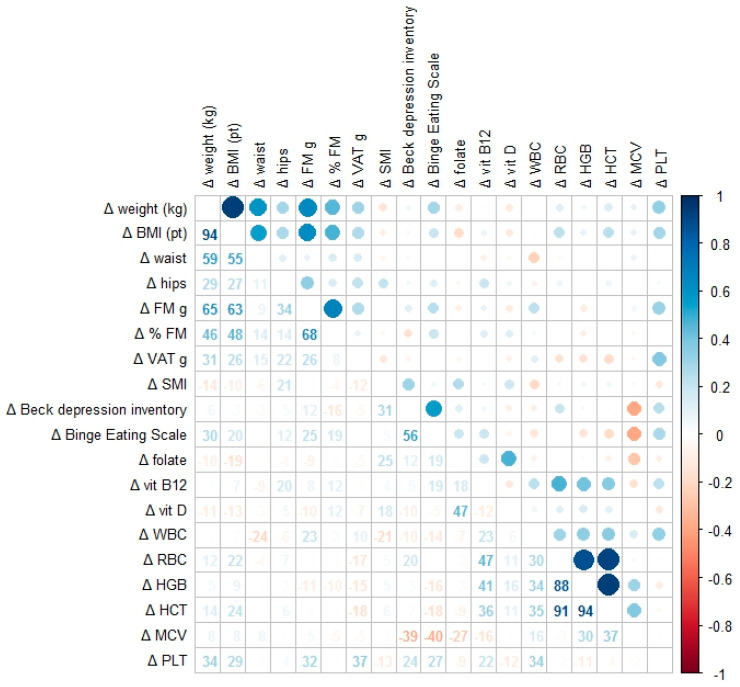
Correlation heatmap showing the associations between changes in anthropometric parameters (Δ weight, Δ BMI (body mass index), Δ fat mass, and Δ VAT), psychological test scores (Δ Beck Depression Inventory and Δ Binge Eating Scale), and hematochemical markers (Δ vitamin D, Δ WBC (white blood cells), Δ RBC (red blood cells), Δ HB (hemoglobin), Δ HCT (hematocrit), Δ MCV (mean corpuscular volume), and Δ PLT (platelets)). Each cell displays the numerical r value and a circle scaled according to the strength of the correlation: blue indicates positive correlations; red indicates negative ones. Only associations with *p* < 0.05 are shown.

The results of the correlation analysis are presented in the heatmap (Figure 1), showing significant associations between changes in anthropometric parameters (Δ weight, Δ body mass index [BMI], Δ fat mass, and Δ visceral adipose tissue [VAT]), psychological test scores (Δ Beck Depression Inventory [BDI] and Δ Binge Eating Scale [BES]), and hematochemical markers (Δ vitamin D, Δ hemoglobin [HB], Δ hematocrit [HCT], Δ white blood cells [WBC], Δ red blood cells [RBC], Δ mean corpuscular volume [MCV], and Δ platelets [PLT]). Only statistically significant correlations (*p* < 0.05) are displayed. The color scale represents Pearson correlation coefficients ranging from −1.0 to +1.0. A moderate positive correlation was observed between reductions in fat mass and improvements in depressive symptoms, with similar correlations noted between changes in BMI and binge eating behaviors.

### 3.1. Psychological Outcomes

Following the multidisciplinary intervention, significant and measurable improvements were observed in all psychological parameters among the participants. These improvements were evident when comparing psychological test scores from baseline to discharge, as detailed in Table 2.

One of the most notable outcomes was the reduction in depressive symptoms. At baseline, 72.3% of participants (129 out of 178) exhibited depressive symptoms above the BDI cut-off of 10. Following the intervention, this percentage dropped significantly to 37.1% (66 out of 178). The mean BDI score decreased from 14.04 (CI95%: 12.32; 15.75) to 8.15 (CI95%: 6.81; 9.49), representing a mean reduction of −5.88 points (CI95%: −7.17; −4.60, *p* < 0.0011). This highlights the substantial alleviation of depressive symptoms achieved through the intervention.

Anxiety levels also improved significantly, as reflected by the State–Trait Anxiety Inventory (STAI) scores. The mean score dropped from 40.25 (CI95%: 36.74; 43.76) at the start of the program to 37.48 (CI95%: 33.84; 41.12) at discharge, corresponding to a mean reduction of −2.77 points (CI95%: −5.02; −0.53, *p* = 0.017).

Similarly, pathological worry, measured using the Penn State Worry Questionnaire (PSWQ), showed significant improvement. The mean score decreased from 47.51 (CI95%: 44.30; 50.73) to 43.17 (CI95%: 39.88; 46.47), with a mean reduction of −4.34 points (CI95%: −7.30; −1.38, *p* = 0.005).

Dysfunctional eating behaviors exhibited the most dramatic improvement, as evidenced by the Binge Eating Scale (BES) scores. The mean BES score dropped significantly from 12.00 (CI95%: 10.30; 13.70) to 5.63 (CI95%: 4.57; 6.70), corresponding to a mean reduction of −6.37 points (CI95%: −7.76; −4.98, *p* < 0.0011).

Body uneasiness, assessed using the Body Uneasiness Test (BUT), also improved slightly but significantly. The mean score decreased from 1.98 (CI95%: 1.21; 2.76) to 1.65 (CI95%: 0.81; 2.50), with a mean reduction of −0.33 points (CI95%: −0.61; −0.05, *p* = 0.023).

These findings underscore the efficacy of the multidisciplinary intervention in reducing psychological distress among obese patients. The results highlight the close interconnection between physical health and mental well-being, affirming the importance of a comprehensive approach that integrates dietary, physical, and psychological strategies in the management of obesity.

### 3.2. Benefits of Multidisciplinary Intervention on Physical, Biochemical, and Psychological Health in Obese Patients

Our study observed significant and concurrent improvements in the physical, biochemical, and psychological parameters of obese patients following a two-month multidisciplinary intervention program. From an anthropometric and body composition perspective, there was a significant reduction in body weight, with an average loss of 7.08 kg and a decrease in BMI of 2.68 kg/m^2^. These changes were accompanied by a notable reduction in waist circumference of 6.35 cm, a critical indicator of visceral adiposity distribution. The reduction in visceral adipose tissue (VAT), estimated at 328.87 g, is particularly significant, as VAT is known to be metabolically active and strongly associated with systemic inflammation and metabolic risks. Simultaneously, we observed a substantial decrease in total fat mass (FM) of more than 4.4 kg, while fat-free mass (FFM) experienced a minor but significant decrease of 1.25 kg. These findings indicate an effective reduction in body weight, predominantly driven by fat mass loss, with a focus on preserving lean mass. This selective reduction is clinically relevant, as preferential fat mass loss is associated with more favorable metabolic and functional outcomes in obese individuals.

In addition to the reduction in adiposity, the improvement in waist circumference suggests a direct positive impact on metabolic risks associated with visceral adiposity, with potential benefits for the patients’ cardiovascular profile. This result is particularly relevant in obese individuals, where VAT plays a critical role in the production of pro-inflammatory cytokines and insulin resistance mechanisms.

From a biochemical perspective, significant increases were observed in serum levels of vitamin D (+14.29 ng/mL), folate (+5.50 ng/mL), and vitamin B12 (+30.48 pg/mL). These micronutrients play a crucial role in key physiological processes such as neurotransmitter synthesis, immune modulation, and the regulation of inflammatory pathways. Their improvement following the intervention suggests an overall enhancement of metabolic health, which is particularly relevant in obese individuals who are frequently affected by micronutrient deficiencies. Notably, the concurrent enhancement of vitamin status and psychological outcomes may reflect a parallel trajectory of metabolic and neuropsychological recovery, although further research is warranted to confirm and better understand this potential association. Finally, the results of psychological tests revealed a significant improvement in depressive symptoms, anxiety, and dysfunctional eating behaviors. Scores on the Beck Depression Inventory (BDI) decreased by an average of 5.88 points, indicating a marked improvement in mood. Anxiety (STAI) scores decreased by 2.77 points, while pathological worry, measured using the PSWQ, showed a reduction of 4.34 points. Binge eating, assessed using the BES, demonstrated a reduction of 6.37 points, indicating better control over binge episodes and improved regulation of eating behaviors. Additionally, a slight but significant reduction in the Body Uneasiness Test (BUT) score of 0.33 points highlighted an improvement in body image perception. These improvements observed across physical, biochemical, and psychological parameters suggest a potentially synergistic benefit of the multidisciplinary intervention, although a causal relationship cannot be inferred from the present data.

### 3.3. Internal Consistency of the Psychological Questionnaire

To evaluate the reliability of the psychological assessment tools used in this study, internal consistency analysis was performed. The psychological evaluation battery was divided into four thematic sections: (1) mood and depressive symptoms, (2) anxiety and worry, (3) eating behavior, and (4) body image perception.

**Figure 2:**
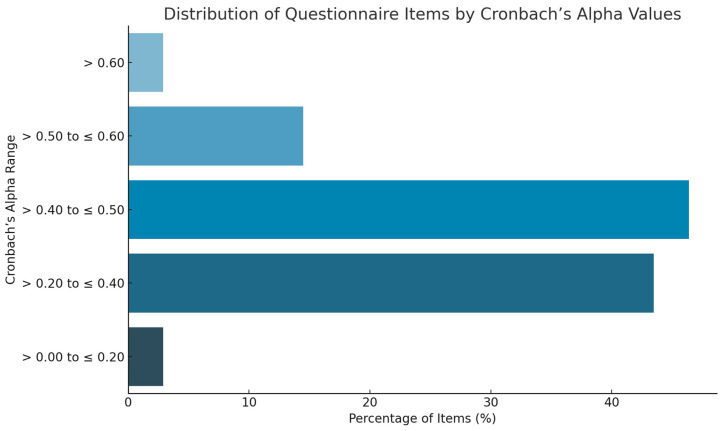
Distribution of questionnaire items according to Cronbach’s Alpha values.

A graphical summary of the distribution of items by Cronbach’s Alpha values is presented in Figure 2 to visually illustrate the internal consistency of the psychological assessment battery

Inter-item correlation scores ranged from 0.08 to 0.53 in Section 1, 0.15 to 0.68 in Section 2, 0.25 to 0.46 in Section 3, and 0.36 to 0.56 in Section 4. The Cronbach’s Alpha values indicated acceptable consistency overall, although some individual items showed lower coherence with the overall scale.

Specifically, two items from Section 1 and Section 2 had Cronbach’s Alpha values between >0.0 and 0.20, representing 2.9% of the questionnaire. A total of 43.48% of the items (30 questions) had values between >0.20 and 0.40, while 46.38% (32 questions) had values between >0.40 and 0.60. Notably, 10 questions (14.49%) achieved values between >0.50 and 0.60, and 2 questions (2.9%) exceeded >0.60 with values up to 0.80.

Overall, 49.28% of the questions had a Cronbach’s Alpha value greater than 0.4, suggesting an acceptable internal consistency. Section 2 (anxiety and worry) contained the highest number of items with lower internal consistency scores (<0.4).

Additionally, some negative values were observed in items 7B and 8A, indicating low coherence of these items with the rest of the scale. These findings suggest that, while the battery overall demonstrates satisfactory internal consistency, certain items may require revision to enhance reliability in future applications.

This figure summarizes the internal consistency analysis of the psychological questionnaire. Items were categorized based on their Cronbach’s Alpha values into five intervals: >0–0.20, >0.20–0.40, >0.40–0.60, >0.60–0.80, and >0.80. The majority of items (49.28%) showed a Cronbach’s Alpha greater than 0.40, indicating acceptable internal reliability. This visual representation supports the results described in the text, highlighting the internal coherence of the questionnaire sections and pinpointing areas that may require refinement in future applications.

## 4. Discussion

The results obtained highlight how a multidisciplinary approach—consisting of dietary, educational, psychotherapeutic, and rehabilitative interventions—acts synergistically on both body composition and psychological health, offering integrated and sustainable benefits in the short term. This strategy allows healthcare professionals to tackle the multifaceted nature of obesity, addressing both physical impairments and underlying psychological challenges in a structured manner. Unlike previous studies, this research uniquely integrates DXA-measured body composition, biochemical markers, and psychological outcomes within a large inpatient cohort, providing a comprehensive real-world evaluation of the effects of such interventions.

Current scientific evidence recognizes that the treatment of obesity requires a multidisciplinary approach involving a team of healthcare professionals, each bringing specific skills to respond to patients’ needs. Physicians address obesity-related medical issues, conducting health assessments and managing comorbidities, while dietitians provide guidance on calorie intake and healthy eating habits. Exercise specialists and physical therapists focus on integrating physical activity into daily routines, developing structured programs to enhance calorie expenditure [32].

In this context, hospitalization provides a controlled environment that ensures patient adherence to treatments and facilitates close monitoring of outcomes. Beyond addressing weight reduction, structured programs like the Body Weight Reduction Program (BWRP) create a foundation for long-term lifestyle changes. Follow-up studies evaluating sustained benefits after discharge are essential to understand the durability of these interventions. Evidence suggests that this combination not only improves body weight but also mitigates obesity-related comorbidities, such as metabolic syndrome and cardiovascular risks [10,33].

The observed improvement in psychological outcomes confirms the association between obesity and poorer mental health outcomes previously highlighted by systematic reviews [34,35]. Our study adds an original contribution by correlating these improvements with simultaneous changes in DXA-measured body composition and biochemical parameters in a large clinical sample. The significant reduction in visceral adipose tissue (VAT) and total fat mass represents a key factor in the psychological improvements observed. VAT, as a metabolically active tissue, is associated with elevated levels of pro-inflammatory cytokines such as IL-6 and TNF-α, which negatively influence mood-regulating neurotransmitters like serotonin and dopamine. This provides a plausible mechanism for the correlation between VAT reduction and the observed decrease in depressive symptoms (BDI score).

Moreover, the recent Mendelian randomization meta-analysis confirmed a causal link between obesity and depression (OR = 1.33), suggesting that weight loss interventions might directly impact mental health risk [36]. The physiological improvements tied to VAT loss extend beyond metabolic benefits, contributing to improved overall functionality and daily performance. Enhanced insulin sensitivity and reduced leptin levels have downstream effects on satiety, energy levels, and mood stability, creating a virtuous cycle that promotes adherence to healthy behaviors. For patients struggling with weight stigma and emotional distress, these changes can mark a turning point in their mental health recovery [37].

Although a minor reduction in fat-free mass (FFM) was observed, the overall improvements in physical and mental well-being offset any adverse effects. Preserving functional muscle mass remains critical to ensuring mobility and independence, particularly in rehabilitation settings where a focus on resistance exercise helps counterbalance FFM loss. Future interventions should incorporate resistance training as a core element to maximize both metabolic and psychological outcomes [38].

The observed increase in vitamin D levels is of interest given its known neuroprotective functions and potential role in mood stabilization, as supported by the existing literature. Recent evidence supports the association between vitamin D deficiency and both depressive and anxiety symptoms in obese individuals, reinforcing the relevance of the observed improvements [39,40,41].

Similarly, increases in folate and vitamin B12 levels may have contributed to supporting neurotransmitter synthesis, neuronal function, and the observed reduction in anxiety (STAI) and pathological worry (PSWQ). Although the design of this study precludes confirmation of causality, these associations are biologically plausible and warrant further investigation in future controlled trials. While these micronutrients are also involved in modulating oxidative stress and inflammatory processes, no inflammatory markers were analyzed or reported in the current analysis.

The significant reductions in BES and BUT scores emphasize the importance of addressing psychological components in obesity management. Systematic reviews have underlined how psychological interventions can improve quality of life, self-efficacy, and adherence in obese patients [42,43]. In some individuals with obesity, patterns of disordered eating behaviors may be present, often influenced by emotional and cognitive factors [44]. The high prevalence of binge eating and emotional eating has been documented, particularly in women [45,46].

Psychological interventions also improve self-awareness and self-regulation skills, empowering patients to identify emotional triggers and adopt healthier coping mechanisms. Group-based therapy fosters a sense of community and shared experience, which can alleviate feelings of isolation and stigma. Reinforcing emotional resilience through tailored psychotherapeutic strategies represents a critical step in achieving long-term behavioral changes that complement physical improvements. The comprehensive integration of anthropometric, biochemical, and psychological data in our study allows for a more holistic understanding of the potential interrelationships, although causality cannot be inferred due to the observational design.

The presence of a support group during interventions proved pivotal, as it facilitated the sharing of common experiences, reduced emotional isolation, enhanced self-efficacy and motivation, and created a positive reinforcement environment that fostered body image improvements and emotional regulation. These findings reinforce the importance of structured psychological support as a cornerstone of multidisciplinary obesity treatment.

### 4.1. Limitation

This study presents several limitations that must be considered when interpreting the results. First, the absence of a control group limits the ability to establish causal relationships between the multidisciplinary intervention and the observed improvements in body composition, biochemical markers, and psychological well-being. Second, the study population consisted exclusively of hospitalized obese patients undergoing an intensive residential program, which may not reflect the broader outpatient or community-based populations and limits generalizability [44]. Third, a potential selection bias exists, since only 178 of the 220 initially screened patients completed the program, potentially leading to an overestimation of treatment effects due to the exclusion of less adherent individuals.

Additionally, although our results are consistent with recent systematic reviews that demonstrate a strong association between obesity and poor mental health outcomes [35,45,46], the observational design of this study does not allow us to determine whether reductions in body fat or improvements in nutritional status were directly responsible for the observed psychological improvements. Mendelian randomization studies have suggested a causal role of obesity in increasing depression risk [36], but further randomized controlled trials are needed to confirm these relationships in clinical settings.

Moreover, another limitation is the sex distribution of the study population, with a higher proportion of female participants compared to males, as well as a significant difference in mean age between sexes. Additionally, the overall mean age of the cohort (58.5 years) reflects an older adult population, which may limit the applicability of findings to younger individuals with obesity.

Finally, internal consistency analysis of the psychological questionnaires revealed some heterogeneity, particularly in the anxiety and worry domain, indicating that future research should consider optimizing or refining the psychometric tools used to assess psychological distress.

Future investigations should include randomized controlled trials with larger and more heterogeneous populations, as well as extended follow-up periods, to verify the long-term efficacy, sustainability, and transferability of multidisciplinary residential interventions for obesity and to better explore the biological and psychological mechanisms underlying the associations observed.

### 4.2. Clinical Applications

The findings of this study support the clinical relevance of structured multidisciplinary residential programs as an effective therapeutic option for the management of severe obesity. Integrating nutritional counseling, supervised physical activity, psychological support, and medical monitoring has been shown to simultaneously improve both physical and psychological health outcomes. Our results are consistent with previous systematic reviews that emphasize the need for combined interventions to address not only weight loss but also the substantial psychological burden of obesity [42,43].

However, the present study adds novel clinical insights by uniquely integrating DXA-measured body composition, detailed biochemical profiling, and validated psychological outcomes within a large real-world inpatient cohort. Unlike previous studies that have often examined these variables in isolation or in outpatient samples [35,45], our study provides a comprehensive evaluation of how improvements in visceral adiposity and metabolic parameters correlate with reductions in psychological distress in a highly controlled clinical setting.

The results confirm that a reduction in visceral adipose tissue and improvements in micronutrient status may be linked to alleviation of depressive symptoms and anxiety, possibly through modulation of inflammatory pathways and neurotransmitter balance [36]. Our findings further corroborate the literature indicating that emotional distress, binge eating behaviors, and weight stigma can be positively influenced through targeted psychological support during weight loss interventions [37].

At the same time, our study does not allow us to definitively confirm a causal relationship between changes in biochemical markers and psychological improvement, highlighting an important area for future research. While consistent with prior evidence, the observational design precludes establishing direct causality [46].

This approach may be particularly valuable for patients with severe obesity and multiple comorbidities who have not responded to outpatient treatments. The controlled residential setting provides continuous professional support, promotes adherence, and reduces exposure to external obesogenic factors. Clinicians are encouraged to consider this model of care, integrating psychological and behavioral interventions into standard obesity management protocols to enhance patient engagement, reduce emotional eating, and mitigate feelings of stigma and isolation [42].

Future clinical applications should aim to refine protocols to optimize fat mass reduction while preserving lean mass, potentially incorporating individualized resistance training alongside dietary and psychological interventions, as recommended in emerging clinical guidelines [38].

## 5. Conclusions

This study highlights how the multidisciplinary approach produced synergistic benefits in obese patients, leading to significant improvements in body composition, biochemical parameters, and mental health. The reduction in visceral fat and waist circumference not only marks metabolic achievements but also indicates reduced cardiovascular risk and systemic inflammation. Additionally, the observed increases in vitamin D, folates, and vitamin B12 further support mood stabilization and cognitive function, directly benefiting depressive and anxiety-related symptoms.

Hospital-based multidisciplinary programs provide a comprehensive care model that successfully addresses both the physiological and psychological dimensions of obesity. These programs deliver immediate results through structured interventions while also establishing behavioral foundations for long-term weight management and mental health recovery. Importantly, studies emphasize the need for post-intervention follow-ups to maintain and monitor progress over time, ensuring sustained improvements.

Moreover, integrating psychological support, structured physical activity, and dietary education allows healthcare professionals to effectively address dysfunctional eating behaviors, stratifying patients based on their relationship with food and emotional triggers. Stratification systems can optimize treatment pathways by distinguishing patients with predominantly hedonic versus homeostatic obesity, facilitating tailored interventions for improved outcomes.

The multidisciplinary approach thus emerges as the most effective strategy for treating obesity, offering benefits that extend far beyond weight loss. By addressing the interplay between metabolic, biochemical, and psychological factors, this integrated model promotes global psychophysical well-being. Moving forward, research efforts should focus on refining these interventions and expanding their accessibility, ensuring that obese individuals receive comprehensive, patient-centered care that supports both their physical and mental health journeys.

Future research should explore the long-term sustainability of the observed improvements, the effectiveness of patient stratification strategies in clinical practice, and the role of individualized protocols incorporating resistance training, micronutrient supplementation, and follow-up psychological support.

In summary, the findings confirm that the multidisciplinary approach not only impacts weight reduction and systemic inflammation but also has a significant effect on mental health and eating behaviors, demonstrating a clear relationship between physical and psychological well-being.

## Figures and Tables

**Table 1:** Descriptive statistics of the sample at baseline: anthropometric parameters, biochemical values, and psychological test.

Variable	Female (Mean ± SD)	Male (Mean ± SD)	Total Sample (Mean ± SD)	*p*-Value
**Anthropometric Parameters**				
Age (years)	61.69 ± 12.511	54.49 ± 15.166	59.10 ± 13.930	<0.001
Height (m)	1.54 ± 0.074	1.70 ± 0.068	1.60 ± 0.160	<0.001
Weight (kg)	99.239 ± 17.448	120.724 ± 18.050	107.024 ± 20.440	<0.001
BMI (kg/m^2^)	41.235 ± 6.522	41.342 ± 5.430	41.274 ± 6.132	0.904
Arm (cm)	37.110 ± 4.770	36.760 ± 4.235	36.981 ± 4.573	0.599
Calf (cm)	41.658 ± 4.869	43.610 ± 3.573	42.360 ± 4.536	0.003
Waist circumference (cm)	119.534 ± 12.539	132.637 ± 11.312	124.259 ± 13.630	<0.001
Hips circumference (cm)	129.366 ± 12.979	124.401 ± 11.515	127.621 ± 12.680	0.008
Total mass (kg)	97.854 ± 16.399	117.195 ± 15.388	104.741 ± 18.505	<0.001
Fat free Mass (g)	46,043.818 ± 6181.026	63,700.521 ± 7650.199	52,331.327 ± 10,817.765	<0.001
Fat mass (g)	48,951.323 ± 107,42.331	50,278.247 ± 103,60.449	49,428.493 ± 10,600.069	0.393
Fat mass (%)	51.456 ± 4.764	43.589 ± 4.829	48.641 ± 6.090	<0.001
FFMI	18,940.226 ± 3232.076	21,525.202 ± 3443.820	19,865.242 ± 3526.936	<0.001
FMI	20,311.478 ± 5273.072	16,990.775 ± 3954.137	19,117.334 ± 5087.413	<0.001
VAT (g)	1971.47 ± 649.296	3343.29 ± 1086.552	2447.57 ± 1052.880	<0.001
SMI	9.052 ± 1.123	10.202 ± 1.029	9.459 ± 1.220	<0.001
Folic acid	7.961 ± 6.587	5.211 ± 2.822	7.031 ± 5.742	0.007
Vitamin B12	383.510 ± 143.929	285.116 ± 121.932	349.373 ± 144.151	<0.001
Vitamin D	19.985 ± 10.748	16.571 ± 10.923	18.776 ± 10.896	0.072
**Biochemical Values**				
WBC	7.054 ± 1.879	7.331 ± 1.818	7.155 ± 1.857	0.305
RBC	4.631 ± 0.474	4.862 ± 0.537	4.715 ± 0.509	0.002
HGB	13.186 ± 1.244	14.295 ± 1.466	13.588 ± 1.429	<0.001
HCT	40.457 ± 3.670	42.626 ± 3.900	41.236 ± 3.887	<0.001
MCV	87.971 ± 4.667	88.288 ± 5.148	88.089 ± 4.841	0.654
PLT	256.120 ± 66.019	248.070 ± 64.959	253.200 ± 65.594	0.397
**Psychological Test**				
BDI	15.980 ± 9.641	11.920 ± 8.460	14.540 ± 9.420	0.005
STAI	42.127 ± 10.176	38.300 ± 10.904	40.905 ± 10.509	0.100
PSWQ	46.760 ± 11.248	43.770 ± 8.854	45.780 ± 10.572	0.205
BES	13.440 ± 9.466	12.220 ± 10.157	13.010 ± 9.707	0.415
BUT	1.845 ± 1.196	2.116 ± 4.783	1.944 ± 3.008	0.706

Abbreviation: BMI: body mass index; FFMI: fat-free mass index; FMI: fat mass index; VAT: visceral adipose tissue; SMI: skeletal muscle index; fat mass (%): percentage of total body weight composed of fat; fat mass (g): total body fat in grams; fat-free mass (g): total lean body mass in grams; WBC: white blood cells; RBC: red blood cells; HGB: hemoglobin; HCT: hematocrit; MCV: mean corpuscular volume; PLT: platelets; BDI: Beck Depression Inventory; STAI: State–Trait Anxiety Inventory; PSWQ: Penn State Worry Questionnaire; BES: Binge Eating Scale; BUT: Body Uneasiness Test.

**Table 2:** Descriptive statistics pre–post-intervention: anthropometric parameters, biochemical values, and psychological test adjusted for age (years), recovery period (days), and gender. In bold: value with *p* < 0.05.

Variable	Baseline Mean (CI95%)	Discharge Mean (CI95%)	Delta Change Pre–Post Mean (CI95%)	*p*-Value
**Anthropometric Parameters**				
Weight (kg)	107.004 (104.525; 109.483)	99.918 (97.650; 102.186)	−7.086 (−7.603; −6.569)	<0.001
BMI (kg/m^2^)	41.339 (40.419; 42.260)	38.653 (37.800; 39.507)	−2.686 (−2.871; −2.501)	<0.001
Arm (cm)	36.729 (35.787; 37.671)	35.166 (34.356; 35.976)	−1.563 (−1.919; −1.207)	<0.001
Calf (cm)	41.885 (41.027; 42.743)	40.805 (40.033; 41.577)	−1.080 (−1.336; −0.824)	<0.001
Waist (cm)	124.375 (122.625; 126.124)	118.029 (116.327; 119.731)	−6.345 (−6.853; −5.837)	<0.001
Hips (cm)	127.803 (125.936; 129.670)	123.187 (121.427; 124.947)	−4.616 (−5.182; −4.050)	<0.001
DXA total mass (kg)	104.186 (101.909; 106.463)	98.466 (96.279; 100.654)	−5.720 (−6.191; −5.250)	<0.001
FFM (g)	52,064.611 (51,123.534; 53,005.689)	50,812.234 (49,950.885; 51,673.584)	−1252.377 (−1591.116; −913.638)	<0.001
FM (g)	49,095.663 (47,473.936; 50,717.389)	44,667.703 (43,096.354; 46,239.052)	−4427.960 (−4817.908; −4038.012)	<0.001
FM (%)	48.393 (47.653; 49.132)	46.386 (45.636; 47.137)	−2.006 (−2.308; −1.705)	<0.001
FFMI	20,014.456 (19,606.832; 20,422.080)	19,435.364 (18,991.582; 19,879.145)	−579.092 (−885.080; −273.105)	<0.001
FMI	19,194.855 (18,510.090; 19,879.619)	17,493.465 (16,845.116; 18,141.813)	−1701.390 (−1855.632; −1547.149)	<0.001
VAT	2437.396 (2309.509; 2565.282)	2108.521 (1990.751; 2226.291)	−328.875 (−400.918; −256.832)	<0.001
SMI	9.431 (9.269; 9.593)	9.289 (9.124; 9.455)	−0.142 (−0.242; −0.042)	0.006
**Biochemical Value**				
Folic acid	7.214 (5.943; 8.486)	12.709 (10.757; 14.662)	5.495 (3.306; 7.684)	<0.001
Vitamin B12	344.157 (317.995; 370.318)	374.637 (347.369; 401.906)	30.480 (10.908; 50.053)	0.003
Vitamin D	18.898 (16.530; 21.266)	33.186 (30.204; 36.169)	14.289 (11.054; 17.523)	<0.001
WBC	7.094 (6.821; 7.366)	6.359 (6.081; 6.637)	−0.734 (−0.907; −0.561)	<0.001
RBC	4.706 (4.634; 4.778)	4.619 (4.551; 4.687)	−0.087 (−0.130; −0.045)	<0.001
HB	13.489 (13.284; 13.693)	13.261 (13.069; 13.453)	−0.228 (−0.348; −0.108)	<0.001
HCT	41.131 (40.548; 41.713)	40.470 (39.906; 41.035)	−0.661 (−1.046; −0.275)	0.001
MCV	88.123 (87.408; 88.838)	88.402 (87.735; 89.070)	0.280 (−0.042; 0.601)	0.088
PLT	250.816 (241.682; 259.950)	231.695 (223.340; 240.051)	−19.121 (−24.351; −13.890)	<0.001
**Psychological Test**				
BDI	14.036 (12.319; 15.752)	8.152 (6.810; 9.494)	−5.884 (−7.166; −4.602)	<0.001
STAI	40.253 (36.743; 43.762)	37.480 (33.844; 41.116)	−2.773 (−5.018; −0.528)	0.017
PSWQ	47.512 (44.297; 50.727)	43.171 (39.877; 46.465)	−4.341 (−7.302; −1.381)	0.005
BES	12.000 (10.302; 13.698)	5.631 (4.566; 6.695)	−6.369 (−7.759; −4.980)	<0.001
BUT	1.981 (1.208; 2.755)	1.653 (0.809; 2.496)	−0.329 (0.611; 0.046)	0.023

Abbreviation: BMI: body mass index; FFMI: fat-free mass index; FMI: fat mass index; VAT: visceral adipose tissue; SMI: skeletal muscle index; fat mass (%): percentage of total body weight composed of fat; fat mass (g): total body fat in grams; fat-free mass (g): total lean body mass in grams; WBC: white blood cells; RBC: red blood cells; HGB: hemoglobin; HCT: hematocrit; MCV: mean corpuscular volume; PLT: platelets; BDI: Beck Depression Inventory; STAI: State–Trait Anxiety Inventory; PSWQ: Penn State Worry Questionnaire; BES: Binge Eating Scale; BUT: Body Uneasiness Test.

## Data Availability

The data presented in this study are available on request from the corresponding author. The data are not publicly available due to privacy restrictions.
